# Automated Cow Body Condition Scoring Using Multiple 3D Cameras and Convolutional Neural Networks

**DOI:** 10.3390/s23229051

**Published:** 2023-11-08

**Authors:** Gary I. Summerfield, Allan De Freitas, Este van Marle-Koster, Herman C. Myburgh

**Affiliations:** 1 Department of Electrical, Electronic and Computer Engineering, University of Pretoria, Pretoria 0028, South Africa; allan.defreitas@up.ac.za (A.D.F.); herman.myburgh@up.ac.za (H.C.M.); 2 Department of Animal Science, University of Pretoria, Pretoria 0028, South Africa; este.vanmarle-koster@up.ac.za

**Keywords:** automated cow body condition scoring, convolutional neural network, computer vision, ensemble modelling, sensor fusion, precision livestock, data augmentation

## Abstract

Body condition scoring is an objective scoring method used to evaluate the health of a cow by determining the amount of subcutaneous fat in a cow. Automated body condition scoring is becoming vital to large commercial dairy farms as it helps farmers score their cows more often and more consistently compared to manual scoring. A common approach to automated body condition scoring is to utilise a CNN-based model trained with data from a depth camera. The approaches presented in this paper make use of three depth cameras placed at different positions near the rear of a cow to train three independent CNNs. Ensemble modelling is used to combine the estimations of the three individual CNN models. The paper aims to test the performance impact of using ensemble modelling with the data from three separate depth cameras. The paper also looks at which of these three cameras and combinations thereof provide a good balance between computational cost and performance. The results of this study show that utilising the data from three depth cameras to train three separate models merged through ensemble modelling yields significantly improved automated body condition scoring accuracy compared to a single-depth camera and CNN model approach. This paper also explored the real-world performance of these models on embedded platforms by comparing the computational cost to the performance of the various models.

## 1. Introduction

Body condition scoring (BCS) is a subjective and non-invasive scoring method used to assess the fat reserves of the dairy cow [1]. The dairy cow has been genetically selected for a high milk yield associated with the mobilization of her body reserves, especially during early lactation. Lactation commences with a loss in body condition, 40–100 days after calving [2], followed by a gradual gain in condition in mid-lactation. Due to the association between the BCS of a cow and milk production, long-term health, and ease of calving [3], it is important to determine and track a cow’s BCS over time to ensure optimal milk production.

Automated BCS is becoming vital to large commercial dairy farms as it assists farmers in scoring their cows more often and more consistently compared to manual methods and scorers [4]. Various existing regression-based and convolutional neural network (CNN)-based approaches to automated cow body condition scoring already exist with some commercial systems also available.

**Figure 1:**
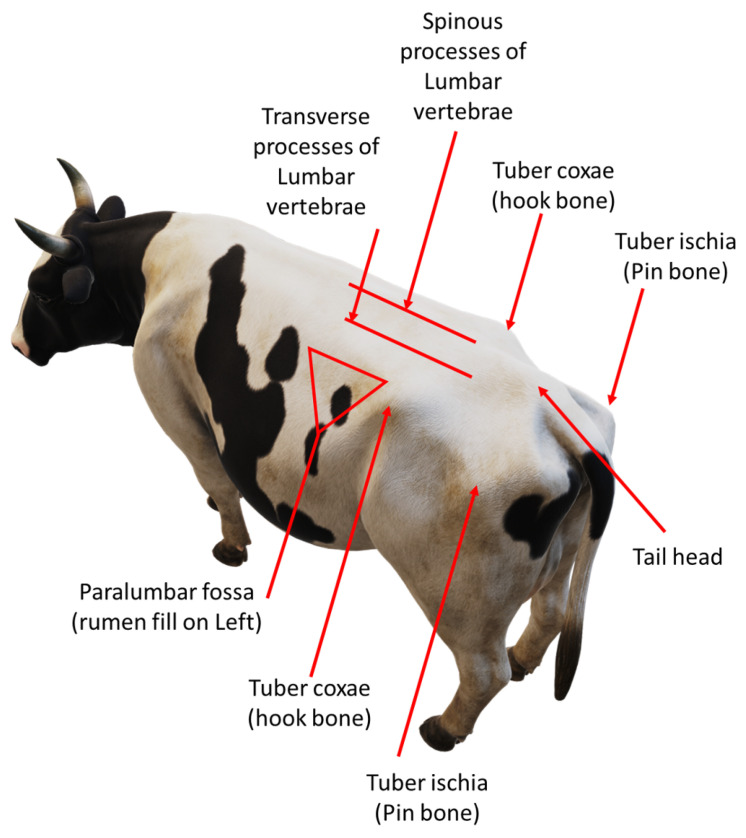
Important anatomical features, on a Holstein cow, used for manual body condition scoring [6].

There are two cow BCS methods, namely the five-point BCS scale and the nine-point BCS scale [4]. The most popular method, and the method used and referenced in this paper, is the five-point scale, which scores cows from 1 to 5 in increments of 0.25. A low BCS value indicates that a cow is under-conditioned while a high BCS value indicates that a cow is over-conditioned. Both of these are undesirable and negatively impact milk production and yield. Most automated cow BCS systems are based on the scoring system derived by Ferguson et al. [5]. The researchers based their scoring system on the work by Edmonson et al. [6]. The main difference between the two is that, in [5], seven anatomical features are used, while in [6], eight are used to score cows. Cow BCS is based on eight anatomical regions, as illustrated in Figure 1. The method described in [6] looks at whether these regions fit certain predetermined profiles. This is primarily performed by looking at how the skin wraps around the bones as well as visually and physically inspecting these regions for how much subcutaneous fat and muscle is under the skin. Usually, overweight cows are more rounded near these regions while underweight cows are more angular due to protruding bones. This indicates how much subcutaneous fat and muscle is built up in these regions and is a visual indicator of how underweight or overweight a cow is.

Automated body condition scoring approaches have yielded very good results in recent years with both regression-based and CNN-based approaches. All CNN-based approaches such as those presented by Alvarez et al. [7], Zhao et al. [8], and Shi et al. [9] made use of a single depth camera placed above the cows. Therefore, research is needed to investigate the use of multiple depth cameras placed at multiple angles to determine whether combining information from multiple cameras can be used to increase the accuracy of an automated BCS system. Multiple-camera approaches such as that presented by Salau et al. [10,11] made use of regression-based approaches and multiple depth cameras placed at multiple angles to improve the accuracy of automated body condition scoring but did not show which camera angles or combinations of cameras yielded the best results.

The novelty of this study is to test the performance impact of using ensemble modelling with the data from three separate depth cameras used to train a variety of CNN models. The study also compares the BCS accuracy of each camera angle model and combinations of these. The study then compares the computational cost to the performance of various approaches for automated body condition scoring.

## 2. Related Research

The research field of automated cow body condition scoring has seen rapid growth in recent years with each year seeing an increase in published works. There are two main approaches, namely regression-based models and neural network-based models [12,13].

### 2.1. Regression-Based Models

Regression-based models involve extracting features from a cow digitally and fitting these features to manually labelled BCS values through various regression techniques. These regression-based models can then be used to estimate the BCS of an unknown cow.

The initial computer-based approach for cow BCS estimation, developed by Bewley et al. [4], involved manually mapping 23 anatomical points to create 15 angles. These angles were used in a first-order auto-regression model to estimate BCS values. The method achieved an accuracy of 99.87% within 0.5 BCS points and 89.95% within 0.25 points of the true BCS value. Although the system lacked real-time operation and automation, it remains one of the most precise semi-automated methods for cow BCS and paved the way for automated BCS systems research.

In contrast to Bewley et al. [4], Halachmi et al. [14] introduced one of the first fully automated real-time regression-based cow body condition scoring (BCS) systems. They used a thermal camera above the cows to capture their contour. The researchers suggested that over-conditioned cows would have rounder contours, resembling a parabolic shape, while under-conditioned cows would differ. They validated their model using both manual and ultrasound-derived BCS values, a method commonly employed by other researchers [15,16]. Hansen et al. [17] also explored a similar concept, introducing a “rolling ball” approach to measure the angularity of a cow’s back surface. This method successfully demonstrated the feasibility of automated real-time BCS using a 3D camera.

One common method for automated BCS is to use depth cameras to extract key anatomical features, and training models to make BCS estimations. Spoliansky et al. [18] developed a model that utilized 14 cow features to determine BCS, with the first 6 related to cow back anatomy and the remainder tied to cow height, weight, and age. This model is different to most as it incorporates non-anatomical features. Using polynomial second-order regression, the model correlated the 14 features with BCS, achieving 74% accuracy within 0.25 BCS, 91% within 0.5 BCS, and 100% within 1 BCS of actual values. This model is claimed to be used in the commercial DeLaval system [19]. Similarly, Song et al. [1] employed three depth cameras to extract information from eight anatomical regions, training a K-nearest neighbour approach model that delivered accurate BCS estimates.

In a study by Zin et al. [20], researchers analysed dairy cows’ backs using 3D surface data, focusing on two regions of interest. The first region involved extracting body condition-related features like average height, convex hull volume, peak and valley point differences, and convex hull volume vs. 3D volume differences. In the second region, they extracted average height and peak-to-valley point differences. Various regression models were developed and compared to estimate BCS based on these features, yielding promising results, despite a small training and testing dataset. Both Zin et al. and Zhao et al. [8] examined the convex hull, with Zin et al. using regression and Zhao et al. employing a neural network approach. Liu et al. [21] defined 3D shape features from six selected cow back regions, using ensemble learning to estimate BCS, achieving favourable results.

### 2.2. Neural Network-Based Models

A newer approach to automated cow BCS is that of using neural networks. Some approaches make use of various techniques to perform feature extraction and use neural networks as classifiers rather than regression. However, some approaches use CNNs to perform both feature extraction as well as classification tasks. Alvarez et al. [13] developed one of the initial automated cow BCS systems using a CNN-based model. It utilized a top-mounted depth camera to capture the back of a cow without extracting specific anatomical features. After the depth image was captured, it was processed to remove unwanted depth noise and isolate only the back of the cow. The input images included depth, edge, and Fourier channels. SqueezeNet [22] was used for the CNN, coupled with data augmentation, resulting in 94% accuracy within 0.5 BCS, 78% within 0.25 BCS, and 40% exact, surpassing automated regression-based approaches. This study highlighted the potential of CNN-based real-time BCS systems for commercial use. The researchers later improved their approach by introducing transfer learning with the VGG16 model [23] and ensemble modelling. While transfer learning lowered the accuracy, the ensemble model achieved 97.4% accuracy within 0.5 BCS, 81.5% within 0.25 BCS, and 41.1% exact [7], representing an enhancement over their previous model [13].

Yukun et al. [16] developed a CNN-based method using ultrasound to measure subcutaneous fat thickness in dairy cows and applied linear regression to match these measurements with manual BCS values. The same ultrasound-based validation was employed in [14,15]. Their regression model served as the reference for training a depth image-based CNN model for automated BCS, with a different architecture (DenseNet) compared to Alvarez et al.’s SqueezeNet.Additionally, Yukun et al. utilized a three-channel approach with depth, grey, and phase congruency data.

Zhao et al. [8] proposed an approach which involved constructing a 3D structure feature map based on the convex hull distance of point clouds, which served as input for a two-level model based on the EfficientNet network. The model yielded an accuracy of 97.6% within 0.5 BCS, 91.2% within 0.25 BCS points, and 45.0% exact. While this approach yielded good results, the model was trained and tested using 5119 depth images of 77 cows, with a 1:1 training–testing dataset ratio. Even though this is a relatively large dataset for this research field, the low number of individual cows from which the dataset originated puts the variation of the dataset into question. This approach is similar to the approach presented in [9], which also made use of point clouds and continued in the direction of neural network-based automated BCS models. The approach used a feature extraction network, which pulled information from 3D point clouds, to extract important features which were then fed into a fully connected neural network trained to estimate the BCS of each cow. The model yielded an accuracy of 96% within 0.5 BCS, 80% within 0.25 BCS points, and 49% exact. To date, this is the most accurate automated cow BCS system which utilises 3D data and neural networks.

Zhao et al. [8] proposed an approach which involved constructing a 3D structure feature map based on the convex hull distance of point clouds, serving as input for a two-level EfficientNet-based model. This model achieved 97.6% accuracy within 0.5 BCS, 91.2% within 0.25 BCS points, and 45.0% exact. However, it was trained and tested on 5119 depth images of 77 cows with a 1:1 training–testing dataset ratio. Even though this is a relatively large dataset for this research field, it raises concerns about dataset variation due to the limited number of individual cows. This approach aligns with the methods presented in [9], utilizing point clouds for neural network-based automated BCS estimation. It involved a feature extraction network to extract crucial features from 3D point clouds, which were then input into a fully connected neural network. This model achieved 96% accuracy within 0.5 BCS, 80% within 0.25 BCS points, and 49% exact, representing the most accurate automated cow BCS system using 3D data and neural networks to date.

All of the CNN-based approaches referenced here have made use of a single-depth camera located above the cows. None of these studies investigated the potential results of capturing depth frames from different angles around the cow. This was one of the goals of the study presented in this paper.

### 2.3. Multiple Camera Approaches

Most automated BCS systems typically use a single overhead camera. However, some systems employ multiple cameras to extract information and enhance automated BCS performance. For instance, Song et al. [1], as discussed in Section 2.1, employed three depth cameras placed above, on the side, and at the rear of the cows. This approach demonstrated the superior information extraction capabilities of multi-camera setups compared to a single overhead camera. Salau et al. [10,11] conducted two studies using six Kinect depth cameras to collect data on the4 linear traits of dairy cows, including teat lengths, ischial tuberosity heights, udder height above ground, and rear leg angles. Both studies employed a specialized frame. Ruchay et al. [24] created an automated computer vision system to produce the precise 3D models of live cattle using data from three Microsoft Kinect™ v2 (Microsoft, Redmond, WA, USA) cameras.

It should be noted that none of these studies used CNNs as the chosen model. Of the multiple camera studies mentioned here, none compared the results of different camera angles or combinations of different camera angles. The study presented in this paper presents the automated BCS results of using various combinations of cameras positioned at different angles around the cows.

### 2.4. Commercial Systems

To date, there are currently three commercially available automated cow BCS systems. These are the DeLaval system from DeLaval International [19], the BodyMat F (BMF) system from Ingenera [25], and the 4DRT-Alpha from Biondi Engineering. Only two of the three have been independently validated. All three systems use 3D image-based approaches.

## 3. Materials and Methods

This paper proposes that the accuracy of CNN-based automated cow body condition scoring can be improved by making use of multiple cameras viewing the cow from different angles and exposing the CNN models to different anatomical features compared to the simple top view used in most approaches.

### 3.1. Data Collection

This study made use of three Microsoft Kinect™ v2 cameras. These cameras allow for the capture of RGB, infrared, and depth images. Only the depth images were captured and used for this study. The RGB and infrared images were not used since the only useful information they provide in this use case is the outline of the cow which can also be produced from a depth image. The depth sensor has a resolution of 512 × 424 and can capture depth images at 30 fps with millimetre precision [26]. The depth images are saved in an array where each pixel value represents the distance from the camera in millimetres.

**Figure 2:**
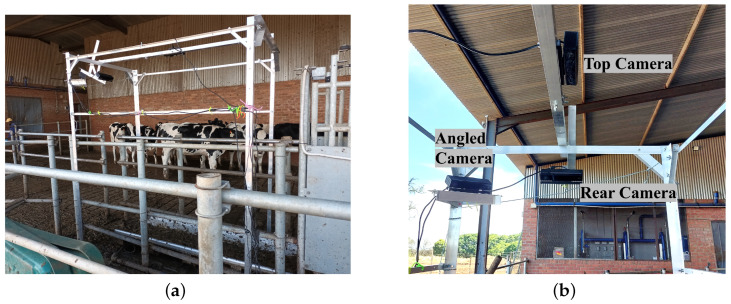
Data collection frame setup: (**a**) Placement of data collection frame over crush; and (**b**) Camera placement on frame.

A camera mounting frame was needed to mount the three cameras and hold them steady while cows passed through the crush. The frame was built in order to be installed over the crush to prevent the cows from bumping the frame or injuring themselves. Figure 2a shows the frame with the cameras standing over the crush where the cows would pass through. The three camera angles that were chosen for this study are a top view, a rear view, and an angled view. These three camera angles captured the most important anatomical regions used for body condition scoring. These anatomical regions can be seen in Figure 1. The rear, top and side regions of the cow are used in body condition scoring and are widely accepted as the most important regions for visually scoring a cow. Figure 2b shows the placement of the three Kinect cameras on the frame.

The system made use of three Jetson Nano™ (Nvidia, Santa Clara, CA, USA) single-board computers to connect to the cameras. This ensured that the system did not encounter any USB bandwidth limits and meant that there was individual control over the cameras. The single-board computers were connected to a laptop using an ethernet LAN with gigabit connections. This setup meant that start and stop commands could be sent to the single-board computers for when to capture depth images. After each stop command, the depth images were sent to the laptop and backed up on an external hard drive.

The data collection for this study took place at a single dairy farm in Rayton near Pretoria, South Africa. The farm has a large dairy parlour in which over six hundred Holstein cows from the host farm and surrounding farms are milked three times a day. This variety meant that there was a large variation in size and body condition score amongst the cows.

**Figure 3:**
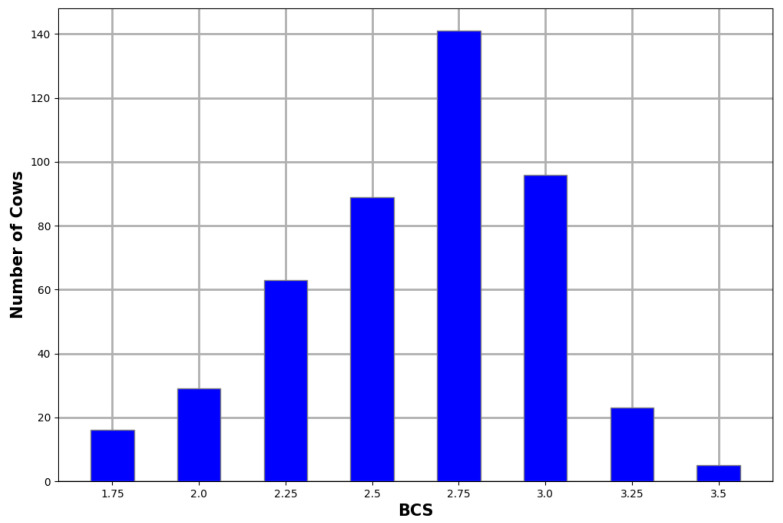
BCS distribution of captured cows.

Two experienced scorers performed the manual body condition scoring on all the cows used in this study according to the five-point BCS system. The distribution of the BCS scores for the animals in this study is illustrated in Figure 3. The scores followed a similar distribution to related studies [7,9,13].

Class imbalance is a common problem in machine learning. Automated body condition scoring research knows this problem all too well with the majority of the training samples sitting in the middle classes and a small number of samples in the edge classes, as can be seen in Figure 3. There are numerous techniques available for dealing with imbalanced classes such as oversampling under-represented classes, undersampling over-represented classes, cost-sensitive learning, and even transfer learning [27]. The technique chosen for this study was cost-sensitive learning and the approach is explained in detail in Section 3.5.3.

Data from a total of 462 cows were used for this study. For the purposes of training the models, approximately 70% of the cows were used for training and 30% were used for testing. Since the Kinect cameras capture at 30 fps, multiple depth frames were captured for each cow that passed the cameras. This helped increase the amount of training data for the models. Since some cows passed quickly and some slowly underneath the camera frame, some cows ended up with a low number of frames captured and some had dozens of frames captured. In order to prevent a large difference in the amount of training data presented to the models from each camera, the number of depth frames per cow was limited to seven. This meant that each cow had at most seven depth frames captured per camera angle. The angled camera captured 2053 depth frames, the rear camera captured 2211 frames, and the top camera captured 2016 frames. A slightly different approach was used for splitting the training and testing data for the CNN models. The typical 70/30 split was performed on the cows and not the overall collected depth frames. In addition, only a single depth frame was used for each cow during testing. This approach was followed to prevent the models from producing the same results for each cow several times over. This meant that, while over 2000 frames were captured for training the models, only 137 unprocessed depth frames were used for testing. Due to data augmentation, which is explained later in this paper, this number was increased to 548.

### 3.2. Image Pre-Processing

**Figure 4:**
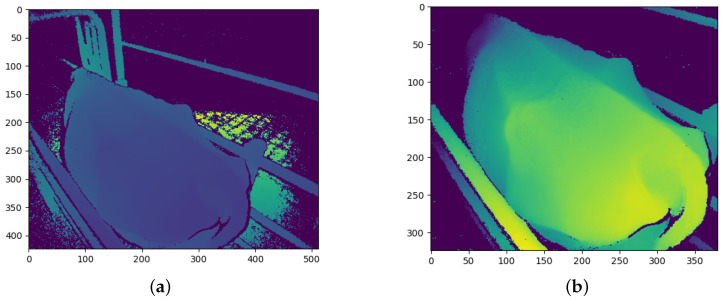
Angled camera depth images before and after pre-processing: (**a**) Before pre-processing; and (**b**) After pre-processing.

**Figure 5:**
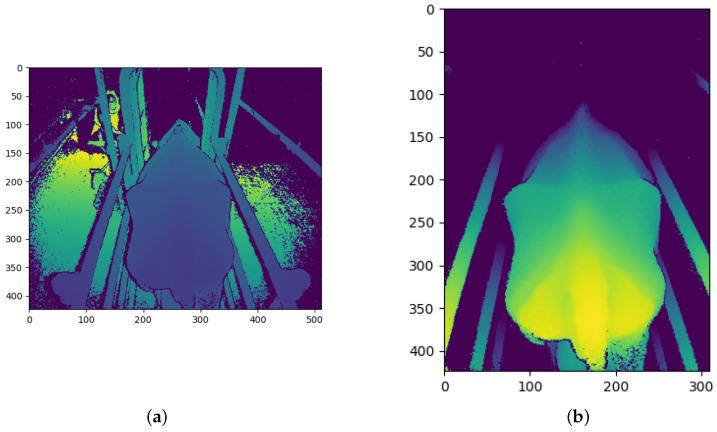
Rear camera depth images before and after pre-processing: (**a**) Before pre-processing; and (**b**) After pre-processing.

**Figure 6:**
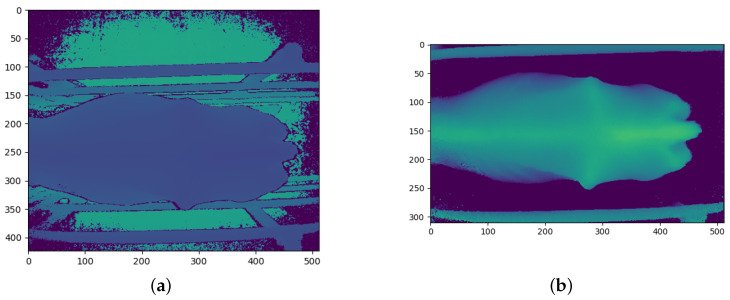
Top camera depth images before and after pre-processing: (**a**) Before pre-processing; and (**b**) After pre-processing.

The Kinect cameras have a good range of between 0.5 m and 4.5 m, in which they can accurately determine the distance to an object [26]. While perfectly suited to this study, the depth images from the cameras contain large amounts of background noise in the form of background objects which may hinder the performance of the CNN models. Before the depth images are processed and converted into the different CNN channels, the background noise must first be removed. Figure 4a shows the raw depth image from the angled Kinect camera. The floor of the inspection pen can be seen behind the cow. The crush can also be seen surrounding the cow. A general approach to removing the background is through background subtraction. Figure 4a, Figure 5a, and Figure 6a show that the cows often cover these background objects, making background subtraction difficult without removing portions of the cow. Looking at the top camera view in Figure 6a, an above-average-sized cow occasionally passes the camera and may touch the top bars of the crush. Therefore, instead of using background subtraction, the depth images were distance-limited, removing any objects which are further than a certain distance from the camera from the image, by zeroing all pixels larger than a certain threshold. The threshold distance was experimentally determined and was chosen to minimise background pixels whilst ensuring that all important anatomical features of the cow are present in the image. Unfortunately, this method does not remove all the background pixels. In principle, the CNN models should learn that these pixels can be ignored and do not affect the BCS of the cows.

Another pre-processing step which was performed is cropping. The depth images were cropped to a specific section of the image, which was found to be ideal for capturing the important regions of the cows as they pass the camera. This region of the image was also determined experimentally. The cropped images are the same size and of the same position in the image for each training and testing sample for each camera. The size and position of the cropped portion of the image are different for each camera, which is why Figure 4b is square, Figure 5b is a vertical rectangle, and Figure 6b is a horizontal rectangle.

The angled camera perspective gives a clear view of the hook bones, pin bones, tail head, rumen-fill, and spinous processes. All of these anatomical regions are crucial to determining the body condition score of a cow. The result of the distance limiting and the cropping for the angled camera is shown in Figure 4b.

The raw depth image from the rear camera has more background noise than the angled camera and can be seen in Figure 5a. The rear camera gives a clear view of the tail head, hook bones, pin bones and spinous processes of the cow. Figure 5b shows the result of the pre-processing of the rear camera depth image.

The top camera angle is the typical camera angle used by many automated body condition scoring studies. Figure 6b shows that most of the important anatomical regions for body condition scoring are visible from the top camera angle. Figure 6a shows that the raw depth image coming from the top camera also contains a substantial amount of background noise and objects. The top camera gives a clear view of the tail head, hook bones, pin bones, and spinous processes of the cow. Figure 6b shows the result of the post-processing of the top camera depth image. Figure 6b shows a fairly small and thin cow which is why there is so much space on either side of the cow.

Even though the three cameras observe similar anatomical regions of the cows, the angles at which these regions are observed are different. This allows the cameras to gather different visual information about each cow which is then presented to the CNN models.

### 3.3. CNN Channel Image Processing

After the depth images were pre-processed, each depth image was transformed to produce two additional images that would be used as additional input channels to the models. The objective is to provide the models with additional information in order for the CNN models to determine which information is most useful. The two transformations that were chosen are binarization and a first derivative filter.

The derivative transform has not been used in previous studies; however, Alvarez et al. [13] made use of the Fourier transform. The derivative transform was chosen with the idea that an over-conditioned cow would produce lower gradient values due to fewer bones protruding and the cow being more rounded whilst an under-conditioned cow would produce larger gradient values due to the protruding of bones leading to larger gradients on the surface of the cow.

The outline of a cow has already been used in previous studies for BCS predictions [4] and has been shown to produce good results. However, the binarized depth image provides the same information but is more pronounced compared to a single thin edge and is likely to be more useful to the CNN model; therefore, binarization is used instead of edge detection. Binarization is an image processing technique in which a set threshold value is used to decide whether a pixel should be set to a one or a zero. Any pixels further away than this threshold are set to 0 and any pixels closer than this threshold are set to 1. The threshold value used in this process was determined experimentally and was chosen such that a silhouette of the cow is formed from the depth image.

The first derivative process calculates the first derivative of the depth image. This process produces an array with the contour gradients over the cow. The gradients were calculated from left to right. Once the gradients were calculated, any gradients above a threshold value were set to zero since some gradients were relatively large along the edge of objects in the depth image compared to the gradients across the body of the cow.

**Figure 7:**
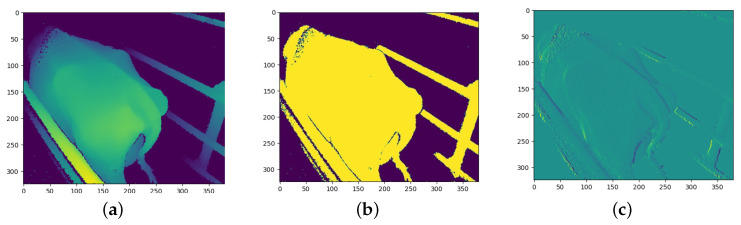
Example of angled camera image processing: (**a**) Depth channel; and (**b**) Binary channel; and (**c**) First-derivative channel.

**Figure 8:**
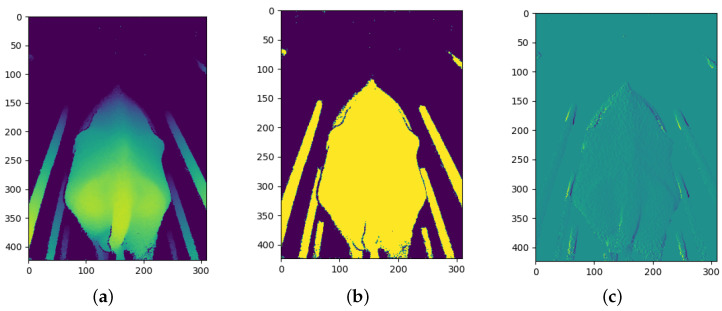
Example of rear camera image processing: (**a**) Depth channel; (**b**) Binary channel; and (**c**) First-derivative channel.

**Figure 9:**
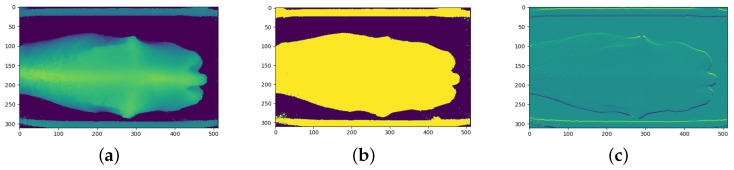
Example of top camera image processing: (**a**) Depth channel; (**b**) Binary channel; and (**c**) First-derivative channel.

Figure 7, Figure 8 and Figure 9 show the three channels used as training data from each of the three cameras.

### 3.4. Data Augmentation

Data augmentation is a common technique used in machine learning to increase the number of training and/or testing samples available by introducing slight changes to these samples [28]. There are many forms of data augmentation. In the field of image-based machine learning, techniques such as geometric transformations, sub-sampling and even filters can be used to slightly change or augment images. In the specific use case of CNN-based BCS models, techniques such as flipping or rotating the images are often used [29]. Unfortunately, rotating the depth image can only be performed if perfect background subtraction has been performed, resulting in only the cow being present in the image. Similarly, flipping the image can only be performed for the top and rear cameras; however, it would not be possible for the angled camera due to the asymmetry. Therefore, the chosen data augmentation technique for this study was sub-sampling. Image sub-sampling, also known as image down-sampling or image decimation, is a technique used in image processing to reduce the size or resolution of an image [30]. It involves reducing the number of pixels in an image while attempting to preserve the important visual information and overall appearance of the image. For this study, each depth image was sub-sampled by a factor of 2, meaning that every second pixel across and every second pixel down was taken to form a new image. This yielded four new images with slightly different information and at half the resolution of the original depth image. This was performed both to increase the number of training samples and to decrease the size of the image being fed into the CNN.

**Figure 10:**
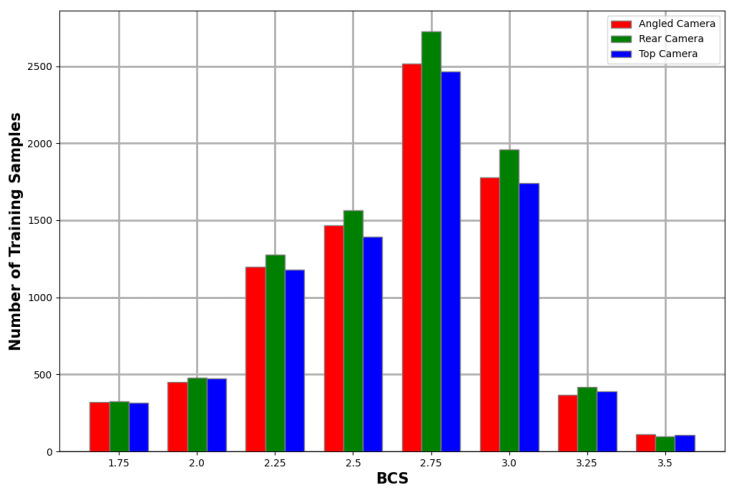
Number of training samples for each BCS values for each camera after data augmentation.

Due to data augmentation, the number of training and testing samples was increased by a factor of four. Table 1 and Figure 10 show the distribution of training data across the different cameras and BCS values.

### 3.5. CNN Models

The approach for this study was to implement and compare the use of multiple camera angles as well as compare various CNN fusion approaches which make use of these multiple camera angles. The rationale is that producing estimations by combining the information from all three cameras will produce a more accurate BCS prediction compared to using a single camera angle since more information is available and processed.

#### 3.5.1. Ensemble Modelling

Ensemble modelling involves the creation of multiple distinct models that work together to produce estimations about a specific outcome. This can be achieved by employing various modelling algorithms or utilizing distinct training datasets [31]. The purpose of ensemble modelling is to increase the overall accuracy of a system compared to using a single model or dataset [32]. Ensemble modelling also helps to reduce model generalisation [32]. This modelling approach is also especially helpful in high-variance models such as CNNs with a large validation loss, where the uncertainty of an individual model is higher than preferred [31].

**Figure 11:**
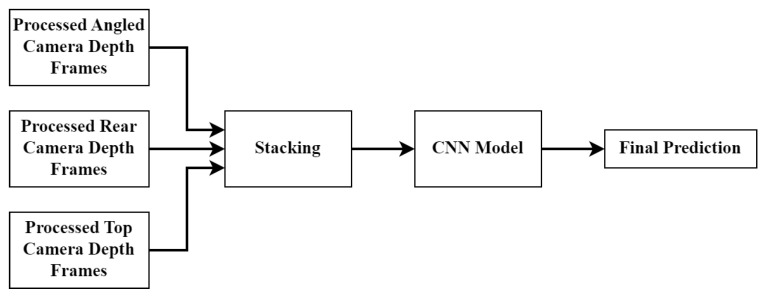
Early fusion CNN model structure.

There are three CNN fusion approaches which were tested in this paper. The first is called the early fusion approach. This architecture effectively stacks the three channels from each camera angle to create a single input consisting of nine channels. The general approach can be seen in Figure 11. Once the nine-channel input is created, the data are fed into a single CNN which produces the final BCS prediction.

**Figure 12:**
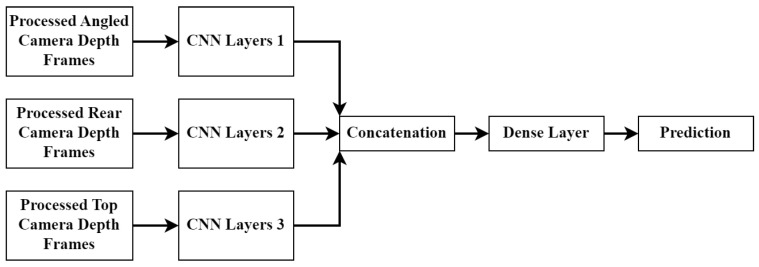
Midof-fion CNN model structure.

The next architecture is called the mid-fusion approach. This architecture involves feeding the data from each camera angle into a set of separate CNN layers. The output from each CNN set is then concatenated and fed into a dense layer network which produces the final prediction. The layout can be seen in Figure 12.

**Figure 13:**
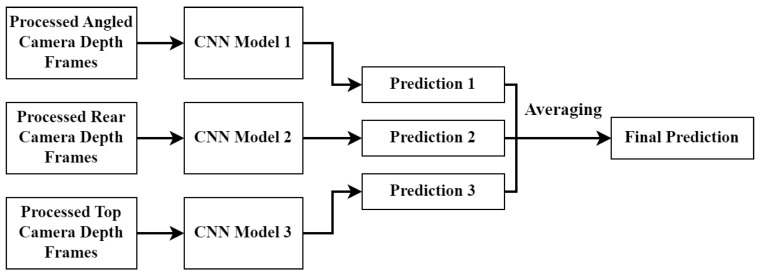
Late fusion CNN model structure.

The final architecture can be seen in Figure 13 and is called the late fusion approach. This architecture consists of three completely separate CNN models (including the dense layers) with the final prediction being the average of the three CNN predictions. Since equal weighting was used for the averaging operation, it was vital to ensure that the number of training samples used for each model remained fairly similar.

The results in Section 4.1 show that, when the three different approaches are trained on the same dataset, the late fusion approach produced the best accuracy results. It is for this reason that the rest of this paper focuses on that approach.

#### 3.5.2. Late Fusion Architecture

**Figure 14:**
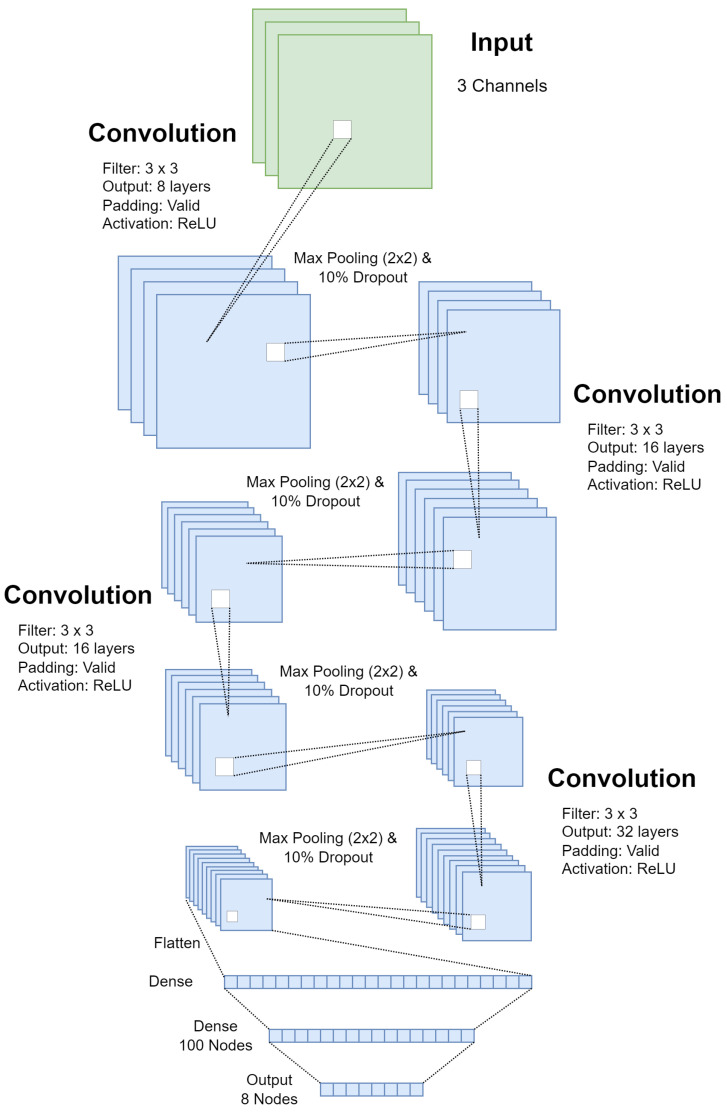
CNN architecture used for all three CNN models in the late fusion approach.

In contrast to the models/architectures proposed in [7,13], a generic CNN with a small number of parameters was chosen for this experiment. The angled, rear, and top CNNs have 265,948, 256,348, and 323,548 trainable parameters, respectively. The generic architecture was chosen since this paper aims to prove that a multi-camera approach to CNN-based BCS models has the capability to improve the overall performance of the combined model. Additionally, an automated BCS system is likely to be run on an embedded platform or a small computer with limited resources; therefore, it is important to focus on smaller and faster BCS prediction models. Figure 14 shows the general CNN architecture which was used for all three camera models. The dimensions for each layer vary for each model due to the differing dimensions of the inputs.

#### 3.5.3. Training

The process of training a CNN model is fairly straightforward and well documented [33,34]. The most important aspects of training CNN models are the architecture and the training and testing data. Both of these were previously discussed in Section 3.2–Section 3.4 and Section 3.5.2. Other important factors in training CNN models include the batch size, the number of epochs used for training and the learning rate. For this study batch sizes of 8, 16, and 32 were, respectively, tested, and there was no clear performance difference. While testing different architectures, the training was often run for 100 epochs. With the dataset being relatively small for training CNNs, the models are prone to overfitting. This is likely why the best-performing models were found between 5 and 30 epochs before overfitting occurs. A learning rate of 0.001 was used for the training. Both larger and smaller learning rates were tested. However, a learning rate of 0.001 seemed to produce the most satisfactory results and was used for the final training.

As mentioned before, there are numerous techniques available for dealing with imbalanced classes such as oversampling under-represented classes, undersampling over-represented classes, cost-sensitive learning, and even transfer learning [27]. Cost-sensitive learning was the chosen technique for this study. Cost-sensitive learning is a technique in which weights are applied to the model loss where the weights are inversely proportional to the number of samples in each class [35,36]. In the case of CNNs, this weighted loss is then applied during backpropagation (training). This technique results in classes with a higher number of training samples affecting the CNN weights less than classes with fewer samples. This prevents the model from becoming class-biased due to a class imbalance in the training data.

### 3.6. Model Performance Evaluation

When evaluating the performance of any model, it is important to use the correct analysis for the specific experiment. In the case of automated body condition scoring, model accuracy and F1-scores are often used. The accuracy is often given in tolerance bands, where the accuracy is calculated by observing how often the model estimates the exact BCS value or estimates within 0.25 or 0.5 of the actual BCS value. Other metrics such as MAE are also often used; however, since the current state-of-the-art generally evaluates the model accuracy, precision, recall, and F1-score, the results in this paper did as well.

Precision is defined as the ratio of true positive predictions to the total positive predictions. In the view of BCS, where multiple classes are present, this can be seen as the ratio of true positive predictions to the total positive predictions for a specific class. For example, when calculating the precision of a model for all cows with a BCS of 3.0, precision is calculated by dividing the number of times the model correctly predicts a cow to be a 3.0 (true positive) by the number of times the model incorrectly predicted a cow to be a 3.0 (false positive).

Recall measures the ratio of true positive predictions to the total actual positives. For example, when calculating the precision of a model for all cows with a BCS of 3.0, recall is calculated by dividing the number of times the model correctly predicts a cow to be a 3.0 (true positive) by the number of times the model incorrectly predicted a cow to have a BCS other than 3.0 (false negative).

The F1-score is the harmonic mean of precision and recall, balancing the trade-off between these two metrics. The goal of the F1-score is to provide a single metric that weights the two ratios (precision and recall) in a balanced way, requiring both to have a higher value for the F1-score value to rise. This means that, if, for example, one model has ten times the precision compared to a second model, the F1-score will not increase ten-fold. The F1-score is a valuable metric for identifying models with both good precision and recall, and since the F1-score can be calculated on a per-class basis, it is possible to see which models have a greater or poorer performance for specific classes.

The accuracy of a typical model can be calculated as

(1) $$Accuracy=\frac{No.\;of\;correct\;Estimations}{No.\;of\;Estimations},$$

and is generally a good indicator of the performance of a model and serves as a good metric to compare various models.

The precision, recall, and F1-score of a model for a single class can be calculated with

(2) $$Precision_{c}=\frac{TP_{c}}{TP_{c}+FP_{c}},$$

(3) $$Recall_{c}=\frac{TP_{c}}{TP_{c}+FN_{c}},$$

(4) $$F1\;score_{c}=\frac{2\times Precision_{c}\times Recall_{c}}{Precision_{c}+Recall_{c}},$$

where TP is the number of true positives, FP is the number of false positives, and FN is the number of false negatives for the model with *c* as the class for which the F1-score is calculated.

Values for precision, recall, and F1-scores are usually calculated for a single class. However, in this paper, the models produce estimations for numerous unbalanced classes. Therefore, instead of producing precision, recall, and F1-score values for each class, a single weighted F1-score can be calculated with the following equation:

(5) $$Weighted\;F1\;Score=\sum_{c}^{N}W_{c}\times F1\;Score_{c},$$

where

(6) $$W_{c}=\frac{No.\;of\;samples\;in\;class\;c}{Total\;number\;of\;samples}.$$

### 3.7. Development Tools

The CNN models were trained using TensorFlow in a Python environment. The hardware that was used included an AMD Ryzen™ (AMD, Santa Clara, CA, USA) 7 5800X 8-Core Processor, 32 GB of memory and a NVIDIA GeForce™ (Nvidia, Santa Clara, CA, USA) RTX 3070Ti GPU with 8 GB of video memory.

## 4. Results and Discussion

This section presents the results of various experiments aimed at testing the performance of a multi-camera BCS approach. These results are discussed in detail as they are presented. The first set of results, as presented in Section 4.1, shows how differing CNN fusion approaches affect the overall performance of the models. Section 4.2 discusses the performance results of the individual cameras while Section 4.3 presents and discusses the performance of the multi-camera approach. Finally, Section 4.4 presents the results of the computational complexity of the various approaches and discusses the findings.

The results in this section present the accuracy of different approaches in three error ranges, namely the exact BCS, within 0.25 BCS, and within 0.5 BCS. The exact error category presents the true accuracy of an approach where the value represents the percentage of testing samples where the approach correctly predicted the BCS of a particular cow. The 0.25 BCS error range represents the percentage of testing samples where the approach predicted the BCS of a cow within 0.25 BCS (one step off) of the actual BCS. Similarly, the 0.5 BCS error range represents the percentage of testing samples where the approach predicted the BCS of a cow within 0.5 BCS (two steps off) of the actual BCS. Veterinarians and other trained cow body condition scorers are often trained to be within 0.25–0.5 of the actual BCS. This is seen as the acceptable human error range [19].

### 4.1. Comparing Different CNN Fusion Approaches

Table 2 shows the accuracy achieved by the different approaches. These approaches were covered in Section 3.5.1. The Late Fusion approach produced the highest accuracy out of the three architectures. It was for this reason that the late fusion approach was elaborated upon in Section 3.5.2.

### 4.2. Individual Camera Model Results

Table 3 shows the accuracy results from each individual CNN trained on the data from a specific camera. The results show that the top camera performed the best for estimating the exact values; however, the angled camera performed the best for estimating the BCS values within 0.25 and within 0.5 of the actual BCS values. These results also indicate that different cameras may lead to better-performing models within certain BCS error ranges compared to others. The table also shows that the three single-camera models follow a similar trend of increasing accuracy as the BCS tolerance increases. The performance of these models does fall below those presented in [7,13] with the reason being the model complexity. However, as stated earlier in this paper, small generic CNN models were chosen since part of the research focus is ensuring that these models are capable of running on embedded platforms.

### 4.3. Ensemble Modelling Results

Table 3 also shows the accuracy results of the ensemble modelling approaches. The first three ensemble models are two-camera combinations of the three individual camera models, namely angled–rear, angled–top, and top–rear. The results show the performances for all three of these two-camera models improved compared to the individual camera models in the exact BCS category. Only the angled camera had a better performance than the top–rear model in the 0.25 and 0.5 BCS error ranges.

Looking at the results of the angled–rear model, the model performed better than either of the individual camera models, improving the accuracies in the exact and within 0.25 tolerance bands by around 2%. The angled–top model, as well as the top–rear model, showed a well-improved performance compared to the performance of the individual camera models, especially when looking at the accuracies achieved estimating the exact BCS values with the top–rear model improving the exact BCS accuracy of the top camera model by 3.28%. These results show that combining the BCS estimation results from two individual camera models has the ability to improve the accuracy of the BCS estimation.

Finally, Table 3 also presents the results of combining the estimations from all three cameras. The all-cameras approach produced the best accuracy results for the exact BCS and 0.25 BCS error range, and also showed high accuracy in the 0.5 error range. Combining the predictions from all three camera models does not always improve the accuracy. However, this is discussed in detail in Section 4.5.

The bold values in Table 3 indicate the highest accuracy values for each tolerance band with the fully combined camera model achieving the best performance in the exact and 0.25 categories. The angled–rear model achieved the best accuracy in the 0.5 category, outperforming the angled camera model due to the performance in the other tolerance bands. An interesting observation is that combining the predictions from multiple camera models increases the accuracy in the exact category; however, the 0.5 category results remain fairly similar across all model combinations. There are several potential reasons as to why the all-camera model does not outperform other models in the 0.5 error range or far outperform other models in the 0.25 error range. One consideration is that the CNNs presented in this study were trained to predict exact BCS values. The cost function used during training makes use of the exact BCS values, which results in the backpropagation altering the weights to minimise this cost according to the exact BCS values. In other words, the model is trained to maximise the accuracy of predicting exact BCS values and not maximise the accuracy of the model within the 0.25 and 0.5 BCS error ranges. Another potential reason is that it is well known that, in sensor fusion, combining too many sources of information may end up hindering the performance of a system. This may be a potential reason for the “all cameras” model not outright outperforming the other models in the 0.25 and 0.5 BCS error ranges.

Table 4, Table 5 and Table 6 show the detailed results of calculating the precision, recall, and F1-score for each of the models for each BCS class. The metric averages were calculated by weighting the values from each class by the number of data samples for each class. The bold values in the class-weighted averages show the best-performing model for each performance measurement.

The results show that, according to these metrics, the combined camera model yielded the best exact BCS performance. However, with the 0.25 error range results, the angled–top model performed the best with the highest F1-score. It is interesting that the model combining all cameras had a lower performance than several other models in this tolerance band. The results are fairly mixed in the 0.5 tolerance band with different models performing the best for each metric. Interestingly, the angled camera model yielded the best F1-score in the 0.5 tolerance band. Again, the model combining all cameras had a lower performance than several other models in this tolerance band.

Overall, the results show that a CNN-based ensemble modelling approach to automated cow BCS using multiple depth cameras generally yields a performance improvement compared to using a single depth camera and single model. However, this seems to only be the case for predicting exact BCS values. If some error is allowed, such as 0.25–0.5 BCS, then single- or two-camera models may be a better choice, especially if equipment cost and computational complexity are a concern.

### 4.4. Computational Complexity Analysis

Since an automated BCS system is expected to operate in the real world and is likely to run on an embedded platform, it is important to consider the computational cost of the various models presented in this study and the real-world implications this may have. Each of the approaches presented in Table 3 was evaluated to examine the relationship between computational complexity and the accuracy achieved by each approach.

**Figure 15:**
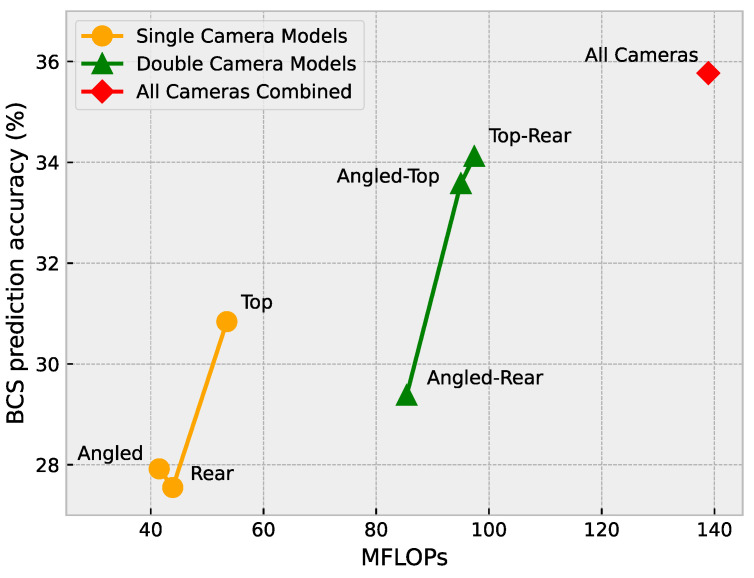
Floating point operations versus BCS prediction accuracy.

Figure 15 shows the number of floating point operations (FLOPs), measured in millions of floating point operations (MFLOPs), for each of the models, and presents the accuracy of each model. From Figure 15, a relationship can be seen where an increase in the FLOPs is mostly followed by an increase in the accuracy. The exception in this case is the rear camera model.

**Figure 16:**
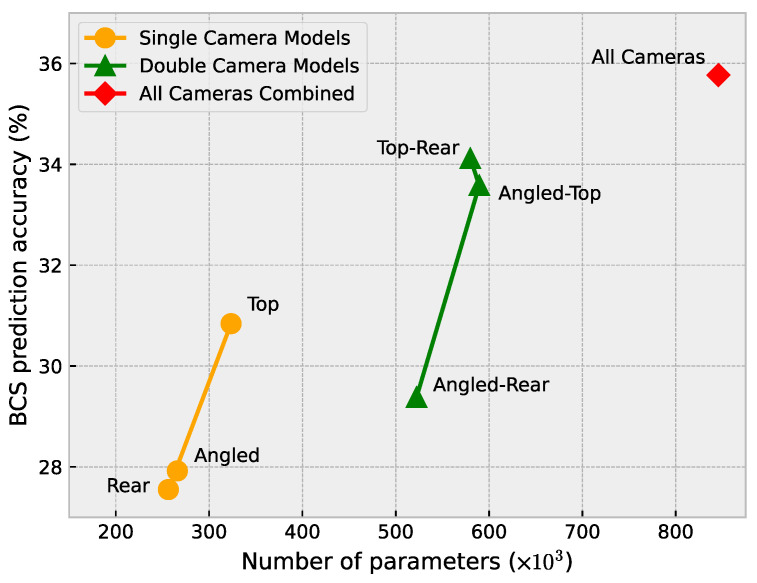
Number of parameters versus BCS prediction accuracy.

Figure 16 shows the number of parameters (CNN weights) for each of the models and also presents the accuracy of each model. A similar relationship to Figure 15 can be seen in Figure 16, where an increase in the number of parameters mostly leads to an increase in the accuracy. The exception in this case is the top–rear model.

**Figure 17:**
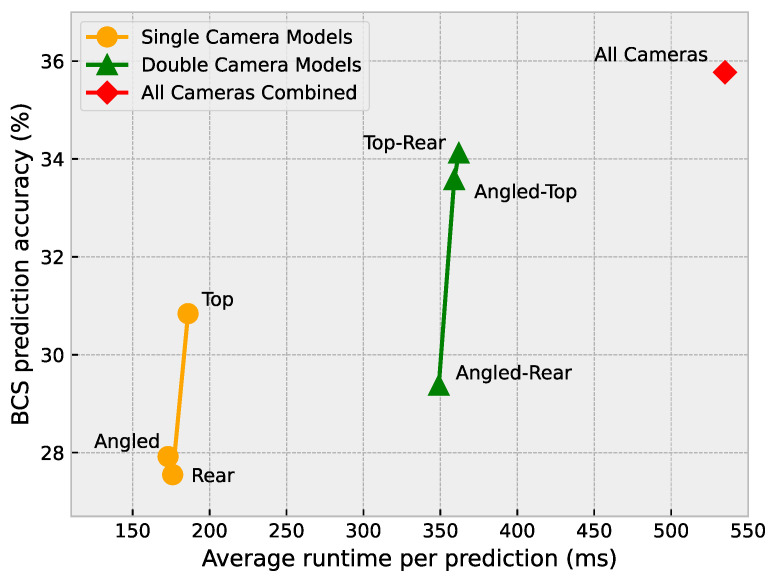
Average runtime per prediction versus BCS prediction accuracy.

Finally, each of the models was run on a Jetson Nano™ with 4 GB of RAM to determine the average time it takes for a single test sample to be processed by each model. Although Figure 17 shows a similar pattern to Figure 15 and Figure 16, the runtimes do not differ greatly within each category of model (single vs. double vs. all cameras). The difference between the angled and top camera models amounts to a mere 13 ms. This can likely be a result of machine learning libraries making efficient use of modern computer architectures. Unless a system’s resources are almost completely used up by a model, the small differences in FLOPS or the number of parameters of each model are almost negligible.

With the current implementation of the all-cameras approach, the model takes around 535 ms on average to process the data from all three cameras and produce a prediction result. During the data collection phase of this study, a cow would pass under the camera frame every 2–5 s. This means that the all-cameras approach, which is the most time-consuming approach, is more than fast enough to generate BCS predictions in real-time on an embedded platform such as a Jetson Nano™.

### 4.5. Noteworthy Observations

During the course of this study, several interesting and noteworthy observations were made.

- Using the three channels as inputs to the CNNs did marginally improve the results compared to only using the depth image as a single channel. However, this was only around 1–2%.
- Data augmentation helped to marginally improve the results compared to only using the original depth images. Using the data-augmented dataset also yielded more consistent training.
- Ensemble modelling performed poorly when the individual camera models had a low validation loss. As expected a large validation loss also yielded poor combined results. A “sweet spot” validation loss of between 3 and 5 produced the best ensemble modelling results on average.
- While combining the three CNN model estimations generally yields an increase in accuracy, this was not always the case. Where the accuracies of the three individual camera models differed significantly, the combined model accuracy might decrease rather than increase. For example, during testing, a certain set of the angled, rear, and top camera models had an exact BCS accuracy of 27%, 28%, and 34%, respectively. The combined model, however, yielded an accuracy of 31%, i.e., meaning that ensemble modelling decreased the accuracy of the top-performing single-camera model. A similar pattern was often observed in other instances where the accuracies of the three individual camera models differed significantly. This showed that it is better in some cases to choose individual models with lower accuracies in order to achieve an increased accuracy for the combined model.

### 4.6. Potential Future Research

When reviewing the work of several researchers in this field, two constant concerns are the low amount of training data combined with the large class imbalance. The researchers in [5] noted that it is difficult to accurately distinguish between BCS values lower than 2 or greater than 4. Therefore, it may be useful to develop a system that classifies a cow with a BCS value below 2 as ”severely under-conditioned” and one above 4 as “severely over-conditioned”. Additionally, this may simplify the models used since there would be fewer classes used for classification and the performance should improve. There would be slightly less class balancing needed.

## 5. Conclusions

This paper performed a novel study investigating the performance gain of an ensemble model approach to automated cow body condition scoring. The proposed approach made use of three depth cameras capturing the depth frames of cows from different angles used to train CNN models. The approach aimed to make use of the extra information provided by the extra camera angles to improve the BCS prediction accuracy compared to a single-camera system. The study aimed to identify which camera angles and which combinations of camera angles increased BCS prediction accuracy. The three-camera approach yielded a significant accuracy improvement compared to a single-camera approach and a double-camera approach. However, single- and double-camera models showed very similar performances to the three-camera approach when 0.25 and 0.5 BCS error ranges were applied.

This paper also explored the suitability of these various approaches when deployed on a real-world embedded platform by comparing the computational cost to the performance of the various models. The general trend was found to be that, as the computational cost increases, the accuracy also tends to increase.

## Figures and Tables

**Table 1:** Number of training and testing samples used to train the final CNN models.

	Angled		Rear		Top	
**BCS**	**Train**	**Test**	**Train**	**Test**	**Train**	**Test**
1.75	320	16	324	16	316	16
2.00	452	44	480	44	472	44
2.25	1196	64	1276	64	1180	64
2.50	1468	128	1564	128	1392	128
2.75	2516	164	2724	164	2464	164
3.00	1780	96	1960	96	1740	96
3.25	368	32	420	32	392	32
3.5	112	4	96	4	108	4
Total	8844	548	8212	548	8064	548

**Table 2:** Model results for estimating body condition scores using different CNN fusion approaches.

Approach	Exact BCS Value (%)	Within 0.25 BCS (%)	Within 0.5 BCS (%)
Early fusion	32.42%	66.33%	**85.04%**
Mid fusion	30.17%	63.34%	82.29%
Late fusion	**34.16%**	**69.08%**	84.54%

Values in **bold** represent the highest accuracies in each category.

**Table 3:** CNN model results for estimating body condition scores using various models.

Approach	Exact BCS Value (%)	Within 0.25 BCS (%)	Within 0.5 BCS (%)
Angled only	27.92%	67.7%	90.15%
Rear only	27.55%	64.6%	85.77%
Top only	30.84%	64.96%	86.86%
Angled–rear	29.38%	69.34%	**90.15%**
Angled–top	33.58%	68.25%	88.87%
Top–rear	34.12%	66.97%	88.32%
All cameras combined	**35.77%**	**69.89%**	89.96%

Values in **bold** represent the highest accuracies in each category.

**Table 4:** Precision, recall, and F1-scores for all models evaluating the exact BCS estimations. The best results are in bold.

BCS	Precision (%)							Recall (%)							F1-Score (%)						
	**A**	**R**	**T**	**A&R**	**A&T**	**T&R**	**All**	**A**	**R**	**T**	**A&R**	**A&T**	**T&R**	**All**	**A**	**R**	**T**	**A&R**	**A&T**	**T&R**	**All**
1.75	0.0	0.0	0.0	0.0	0.0	0.0	0.0	0.0	0.0	0.0	0.0	0.0	0.0	0.0	0.0	0.0	0.0	0.0	0.0	0.0	0.0
2.00	23.08	0.0	48.48	16.13	44.44	40.0	48.28	27.27	0.0	36.36	11.36	27.27	22.73	31.82	25.0	0.0	41.56	13.33	33.8	28.99	38.36
2.25	8.57	29.41	5.0	0.0	1.96	5.45	5.0	4.69	15.62	6.25	0.0	1.56	4.69	4.69	6.06	20.41	5.56	0.0	1.74	5.04	4.84
2.50	34.19	27.15	37.5	34.78	46.27	38.46	41.1	31.25	46.88	16.41	50.0	24.22	27.34	23.44	32.65	34.38	22.83	41.03	31.79	31.96	29.85
2.75	35.8	31.71	35.86	30.26	35.71	38.18	37.18	35.37	31.71	54.88	35.98	57.93	64.02	62.8	35.58	31.71	43.37	32.87	44.19	47.84	46.71
3.00	24.66	42.62	36.54	30.28	36.89	43.59	42.86	37.5	27.08	39.58	34.38	46.88	35.42	40.62	29.75	33.12	38.0	32.2	41.28	39.08	41.71
3.25	12.5	0.0	0.0	0.0	0.0	0.0	0.0	12.5	0.0	0.0	0.0	0.0	0.0	0.0	12.5	0.0	0.0	0.0	0.0	0.0	0.0
3.50	0.0	37.5	0.0	0.0	0.0	0.0	0.0	0.0	75.0	0.0	0.0	0.0	0.0	0.0	0.0	50.0	0.0	0.0	0.0	0.0	0.0
Class Weighted Average	26.60	27.00	30.37	23.78	31.75	31.90	**32.70**	27.92	27.55	30.84	29.38	33.58	34.12	**34.49**	26.93	26.07	28.95	26.13	30.80	31.54	**31.90**

**Table 5:** Precision, recall, and F1-scores for all models evaluating the estimations within 0.25 BCS. The best results are in bold.

BCS	Precision (%)							Recall (%)							F1-Score (%)						
	**A**	**R**	**T**	**A&R**	**A&T**	**T&R**	**All**	**A**	**R**	**T**	**A&R**	**A&T**	**T&R**	**All**	**A**	**R**	**T**	**A&R**	**A&T**	**T&R**	**All**
1.75	0.0	0.0	0.0	0.0	0.0	0.0	0.0	0.0	0.0	0.0	0.0	0.0	0.0	0.0	0.0	0.0	0.0	0.0	0.0	0.0	0.0
2.00	51.06	30.43	56.25	42.86	60.71	58.33	53.85	54.55	15.91	40.91	27.27	38.64	31.82	31.82	52.75	20.9	47.37	33.33	47.22	41.18	40.0
2.25	66.04	77.27	24.62	74.29	33.33	37.5	32.65	54.69	53.12	25.0	40.62	23.44	23.44	25.0	59.83	62.96	24.81	52.53	27.52	28.85	28.32
2.50	67.42	58.05	88.79	64.85	87.72	79.51	86.09	69.53	78.91	74.22	83.59	78.12	75.78	77.34	68.46	66.89	80.85	73.04	82.64	77.6	81.48
2.75	77.65	73.54	63.37	71.36	62.95	62.08	61.04	80.49	84.76	78.05	92.68	85.98	90.85	85.98	79.04	78.75	69.95	80.64	72.68	73.76	71.39
3.00	64.55	81.01	78.79	80.68	78.22	87.64	86.02	73.96	66.67	81.25	73.96	82.29	81.25	83.33	68.93	73.14	80.0	77.17	80.2	84.32	84.66
3.25	58.82	50.0	100.0	85.71	100.0	100.0	100.0	62.5	18.75	65.62	37.5	65.62	40.62	56.25	60.61	27.27	79.25	52.17	79.25	57.78	72.0
3.50	0.0	42.86	0.0	0.0	33.33	50.0	0.0	0.0	75.0	0.0	0.0	25.0	25.0	0.0	0.0	54.55	0.0	0.0	28.57	33.33	0.0
Class Weighted Average	65.54	64.46	66.73	67.76	**67.88**	67.77	67.42	67.70	64.60	64.96	**69.34**	68.25	66.97	67.15	66.48	63.03	65.16	66.57	**66.95**	65.26	65.95

**Table 6:** Precision, recall, and F1-scores for all models evaluating estimations within 0.5 BCS. The best results are in bold.

BCS	Precision (%)							Recall (%)							F1-Score (%)						
	**A**	**R**	**T**	**A&R**	**A&T**	**T&R**	**All**	**A**	**R**	**T**	**A&R**	**A&T**	**T&R**	**All**	**A**	**R**	**T**	**A&R**	**A&T**	**T&R**	**All**
1.75	0.0	0.0	18.18	0.0	20.0	18.18	21.05	0.0	0.0	25.0	0.0	18.75	25.0	25.0	0.0	0.0	21.05	0.0	19.35	21.05	22.86
2.00	80.0	78.26	68.75	80.0	84.62	86.67	77.78	81.82	81.82	50.0	72.73	50.0	59.09	47.73	80.9	80.0	57.89	76.19	62.86	70.27	59.15
2.25	96.36	92.06	86.49	98.39	93.85	90.14	90.14	82.81	90.62	100.0	95.31	95.31	100.0	100.0	89.08	91.34	92.75	96.83	94.57	94.81	94.81
2.50	90.07	84.4	100.0	85.91	99.15	97.41	99.13	99.22	92.97	86.72	100.0	90.62	88.28	89.06	94.42	88.48	92.89	92.42	94.69	92.62	93.83
2.75	92.57	90.74	88.59	89.01	86.32	84.46	83.59	98.78	89.63	99.39	98.78	100.0	99.39	99.39	95.58	90.18	93.68	93.64	92.66	91.32	90.81
3.00	85.58	96.77	90.53	95.83	87.13	98.88	95.65	92.71	93.75	89.58	95.83	91.67	91.67	91.67	89.0	95.24	90.05	95.83	89.34	95.14	93.62
3.25	100.0	85.0	100.0	100.0	100.0	100.0	100.0	84.38	53.12	81.25	59.38	100.0	78.12	87.5	91.53	65.38	89.66	74.51	100.0	87.72	93.33
3.50	0.0	100.0	0.0	0.0	50.0	50.0	0.0	0.0	75.0	0.0	0.0	25.0	25.0	0.0	0.0	85.71	0.0	0.0	33.33	33.33	0.0
Class Weighted Average	87.25	86.55	87.72	87.25	88.80	**89.57**	88.15	90.15	85.77	86.86	**90.15**	88.87	88.32	87.96	**88.49**	85.87	86.84	88.18	88.24	88.32	87.43

## Data Availability

The data presented in this study are available upon request from the corresponding author. The data are not publicly available due to privacy concerns.

## Abbreviations

The following abbreviations are used in this manuscript:

BCS	Body Condition Score/Scoring
CNN	Convolutional Neural Network
FLOPs	Floating Point Operations
MAE	Mean Absolute Error
MFLOPs	Millions of Floating Point Operations
RGB	Red–Green–Blue
